# The cGAS-STING pathway-related gene signature can predict patient prognosis and immunotherapy responses in prostate adenocarcinoma

**DOI:** 10.1097/MD.0000000000031290

**Published:** 2022-12-16

**Authors:** Xingxing Zhuo, Hao Dai, Sui Yu

**Affiliations:** a Department of Urology, Fenghua District People’s Hospital, Ningbo, People’s Republic of China.

**Keywords:** cGAS, immune therapy, prognosis, prostate adenocarcinoma, STING

## Abstract

The cyclic GMP-AMP synthase-stimulator of the interferon genes (cGAS-STING) pathway is essential in inflammation-driven tumor occurrence and progression. However, the prognostic roles and immune functions of cGAS-STING pathway-related genes in patients with prostate adenocarcinoma (PRAD) remain unclear. cGAS-STING pathway-related genes were obtained from the gene set enrichment analysis (GSEA) website. Univariate Cox regression analysis was performed to screen the prognosis-related hub genes in the cancer genome atlas (TCGA) and GSE116918 datasets. Unsupervised clustering analysis was performed to identify different clusters. The least absolute shrinkage and selection operator and multivariate Cox regression analyses were applied to develop a prognostic risk model. The prognostic values and predictive performance of risk signature were assessed by the Kaplan–Meier curve and receiver operating characteristic curve. The IMvigor210 cohort was used to investigate the potential values of the risk score in immunotherapeutic responses. Two clusters were identified based on the expression matrix of 12 prognosis-related genes. Specifically, better overall survival was observed in cluster 2 than cluster 1 in both datasets. Inflammation-related pathway enrichment and immune cell infiltration levels were altered between 2 clusters. Moreover, 6 genes (*CASP8*, *GRK6*, *IL3RA*, *PLCB1*, *TBKBP1*, and *TNFSF10*) were identified to generate a cGAS-STING pathway-related signature (CPRS). Survival analysis showed that patients in the high-risk group showed a more dismal survival than those in the low-risk group in TCGA and GSE116918 datasets. Notably, the CPRS can differentiate responsive patients from non-responsive individuals treated with PD-L1 blockades in an independent cohort. In addition, higher CPRS was associated with a more favorable prognosis. The proposed risk model was developed based on 6 cGAS-STING pathway related-genes, which can be used as a promising predictor for patient survival and immunotherapeutic responses in PRAD, contributing to treatment strategy-related decision-making.

## 1. Introduction

Prostate cancer is one of the common malignant tumors in men, and its morbidity and mortality rates rank second and fifth worldwide, first and third in Europe and America, and sixth and seventh in China, respectively.^[1]^ Radical prostatectomy or radiation therapy is the primary curative therapy for prostate cancer patients. However, approximately 20% to 30% of patients will suffer from biochemical recurrence after radical prostatectomy, which is an initial event of recurrence and metastases.^[2,3]^ Currently, accurate prediction of patient survival risk is still challenging. Although some indicators, such as serum prostate-specific antigen, pathological tumor stage, and Gleason score, have been proven effective predictors,^[4]^ the prediction performance still needs improvement.

Cyclic GMP-AMP synthase (cGAS) is a DNA sensor, and it can recognize cytoplasmic DNA and trigger downstream stimulator of interferon genes (STING).^[5,6]^ Disfunction of the cGAS-STING pathway is associated with tumorigenesis and cancer progression, while activation of this pathway can induce adaptive anti-tumor immune responses.^[5,6]^ A pan-cancer study demonstrated that a high expression level of cGAS-STING signaling was found in pan-cancer tissues and was associated with dismal prognosis in patients with some cancer types,^[7]^ suggesting the significant values of cGAS-STING signaling in tumor progression. A recent study indicated that a risk model generated by 5 cGAS-STING pathway-related genes (*IFNB1*, *IFNA4*, *IL6*, *NFKB2*, and *TRIM25*) could predict overall survival (OS) in patients with gastric cancer.^[8]^ However, the prognostic values of cGAS-STING pathway-related genes in prostate adenocarcinoma (PRAD) are still unclear.

This study used bioinformatics methods to construct a cGAS-STING pathway-related signature (CPRS) in patients with PRAD. The predictive performance of CPRS was validated in an independent cohort. In addition, the values of CPRS in assessing immuno-therapeutic responses were identified based on the IMvigor210 dataset.

## 2. Materials and Methods

### 2.1. Data collection and processing

The mRNA expression matrix, clinicopathological data, survival information, and somatic mutational profile of patients with PRAD were downloaded from The Cancer Genome Atlas (TCGA) (https://gdc.cancer.gov/). In addition, the expression information and clinicopathological data of patients with prostate cancer were also obtained from an independent dataset (GSE116918).^[9]^ Patients with missing data were excluded for subsequent analysis. Normalization was performed for all data used in this study.

### 2.2. Extraction of cGAS-STING pathway-related genes

The cGAS-STING pathway-related genes were identified based on the GSEA database (http://www.gsea-msigdb.org/gsea/index.jsp) in TCGA and GSE116918 datasets. In addition, univariate Cox regression analysis was performed to screen the prognosis-related hub genes, with the *P* value < .05. Intersection analysis and Venn diagram were used to screen the common prognostic genes between the 2 datasets.

### 2.3. Consensus clustering analysis

To further classify patients, unsupervised consensus clustering analysis was performed based on the expression levels of identified prognostic genes after intersection analysis *via* the R package of “ConsensusClusterPlus” (clusterAlg = pam, distance = canberra). The definition of the optimal number of clusters is the k value where the magnitude of the cophenetic correlation coefficient starts to decrease. Survival analysis and Gene Set Enrichment Analysis (GSEA) were conducted between clusters. The gene set of “h.all.v7.5.entrez.gmt” was used when performing GSEA, and the R package of “clusterProfiler” was applied in this step.

### 2.4. Construction and assessment of a risk model

The least absolute shrinkage and selection operator Cox regression analysis was utilized to select the most relevant prognostic genes.^[10,11]^ The CPRS was generated based on the expression levels of identified gene and the corresponding least absolute shrinkage and selection operator Cox coefficients. Then, patients were divided into high- and low-risk groups based on the median cutoff value of CPRS. The predictive power of CPRS was assessed by the survival analysis and time-dependent receive operating characteristic curves in the TCGA dataset. Then it was verified in the GSE116918 dataset. Additionally, univariate and multivariate Cox regression analyses were performed to identify the prognostic values of CPRS in the TCGA dataset.

### 2.5. Immune infiltration levels and immune-therapeutic response assessment

The ESTIMATE algorithm was applied to generate the StromalScore and ImmuneScore, representing the presence of stromal and immune cells. To assess tumor purity, the ESTIMATEScore was obtained after integrating with both scores. Distribution differences of 3 scores were compared between the high-risk and low-risk groups in the TCGA dataset, then validated in the GSE116918 dataset. In addition, the distribution levels of 22 immune cells were also identified in both groups.

To further detect potential values of CPRS in the assessment of immune-therapeutic response, an independent cohort (IMvigor210) was used for subsequent analysis.^[12]^ The IMvigor210 cohort included the expression profile and clinical information of responded or non-responded patients with metastatic urothelial cancer who received anti-PD-L1 agents. In addition, Kaplan–Meier curves were drawn to identify the prediction performance of CPRS.

### 2.6. Statistical analysis

Continuous variables were tested by the Mann–Whitney or Student *t* tests appropriately. Chi-square or Fisher’s exact tests examined categorical variables as appropriate. Survival analysis was assessed by the Kaplan–Meier method and compared by the log-rank test. The Benjamini and Hochberg correction was used to adjust the *P* values in this step. The R software (version 3.6.2; https://www.r-project.org/) was used for statistical analyses. *P *< .05 was considered as statistical differences.

## 3. Results

### 3.1. Identification of 12 prognosis-related genes

**Figure 1.**
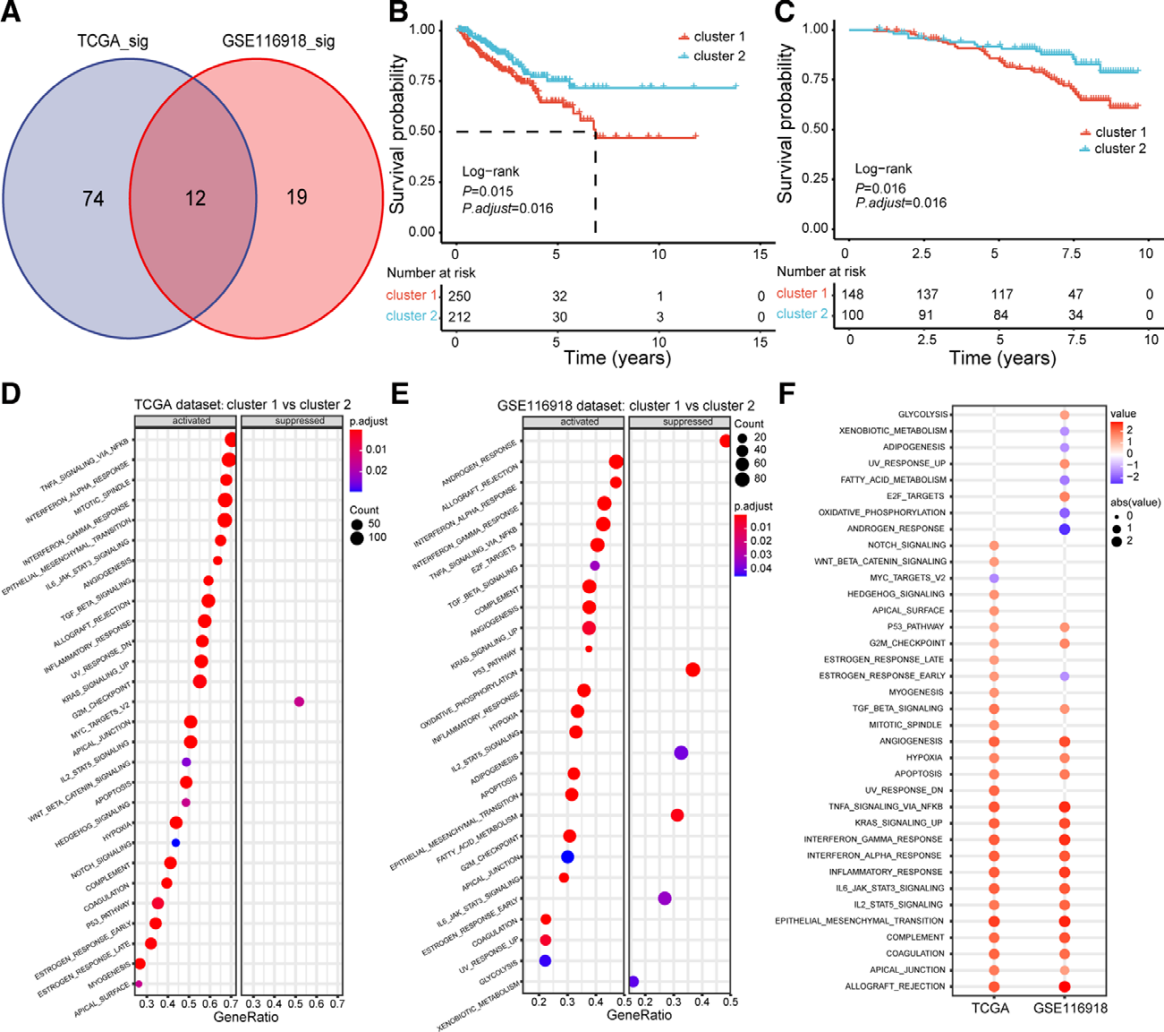
Survival and enrichment pathway analyses of cGAS-STING pathway-related gene clusters based on the unsupervised clustering analysis. (A) Venn diagram of cGAS-STING pathway-related hub genes in the TCGA and GSE116918 datasets. (B and C) Survival analysis of different cluster by Kaplan–Meier curves in the TCGA and GSE116918 datasets, respectively. (D and E) Enrichment pathway analyses between the cluster 1 and cluster 2 in the TCGA and GSE116918 datasets, respectively. (F) Intersection analysis of enrichment pathways between the TCGA and GSE116918 datasets. cGAS-STING = The cyclic GMP-AMP synthase-stimulator of interferon genes, TCGA = the cancer genome atlas.

Three hundred twenty-six genes were obtained from the GSEA website and identified in both databases in this study. Univariate Cox regression analysis showed 86 and 31 prognosis-related genes in TCGA and the GSE116918 databases, respectively (Fig. 1A). Intersection analysis identified 12 genes as common indicators associated with patient survival between the 2 datasets (Fig. 1A).

### 3.2. Identification of molecular subtypes in PRAD

Two clusters were defined according to the consensus clustering analysis according to the profiles of twelve hub genes (Fig. S1A and B, http://links.lww.com/MD/H699). Survival analysis demonstrated that patients in cluster 2 had a more favorable prognosis than cluster 1 in the TCGA cohort (*P* = .015, Fig. 1B). A similar survival tendency was validated in the GSE116918 cohort (*P* = .016, Fig. 1C).

GSEA analysis demonstrated that cluster 1 was positively correlated with inflammation-related pathways in the TCGA dataset (Fig. 1D), such as TNFA_SIGNALING_VIA_NFKB, INTERFERON_ALPHA_RESPONSE, INTERFERON_GAMMA_RESPONSE, EPITHELIAL_MESENCHYMAL_TRANSITION, IL6_JAK_STAT3_SIGNALING, etc. Similarly, inflammation-associated pathways were also validated in the GSE116918 dataset (Fig. 1E and F).

**Figure 2.**
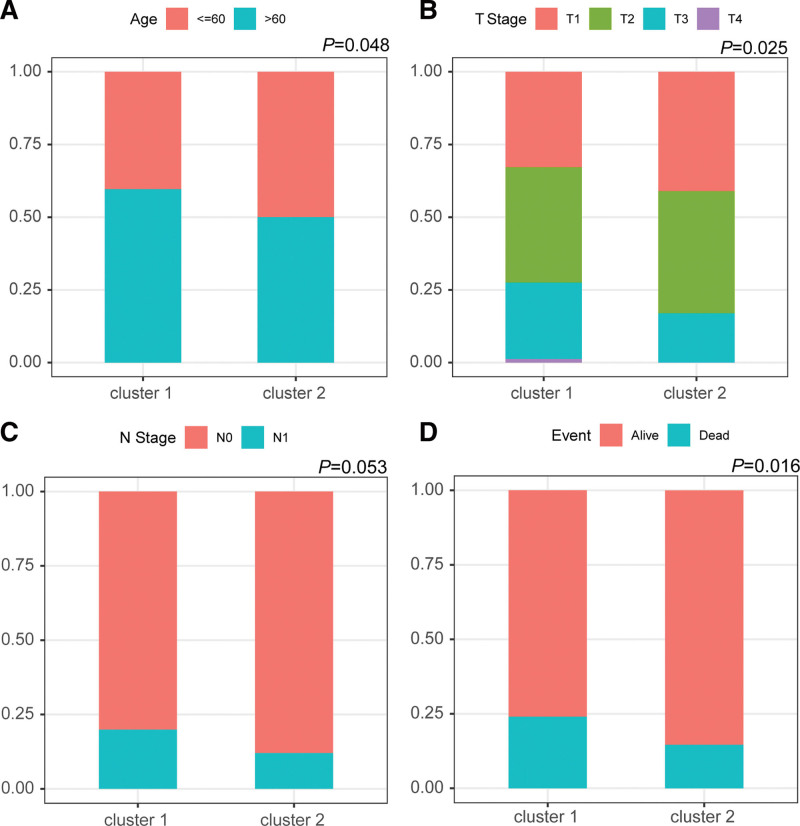
Comparisons of percentage of different clinical characteristics between the cluster 1 and cluster 2. (A) The distribution rates of patients with different ages in the cluster 1 and cluster 2. (B) The distribution rates of patients with different T stages in the cluster 1 and cluster 2. (C) The distribution rates of patients with different N stages in the cluster 1 and cluster 2. (D) The distribution rates of patients with different survival stages in the cluster 1 and cluster 2.

Moreover, we further compared the differences in clinical characteristics of patients in 2 clusters. The results showed that diversity patterns between clusters 1 and 2 were detected regarding patient age, T stage, N stage, and survival comparison (Fig. 2A–D). The percentage of patients younger than 60 years old was higher in cluster 2 than in cluster 1 – higher proportions of patients with T1 or N0 stage in cluster 2. Better tumor characteristics were shown in cluster 2 compared with those in cluster 1, contributing to more favorable survival.

### 3.3. Immune microenvironment (TME) in the 2 clusters

**Figure 3.**
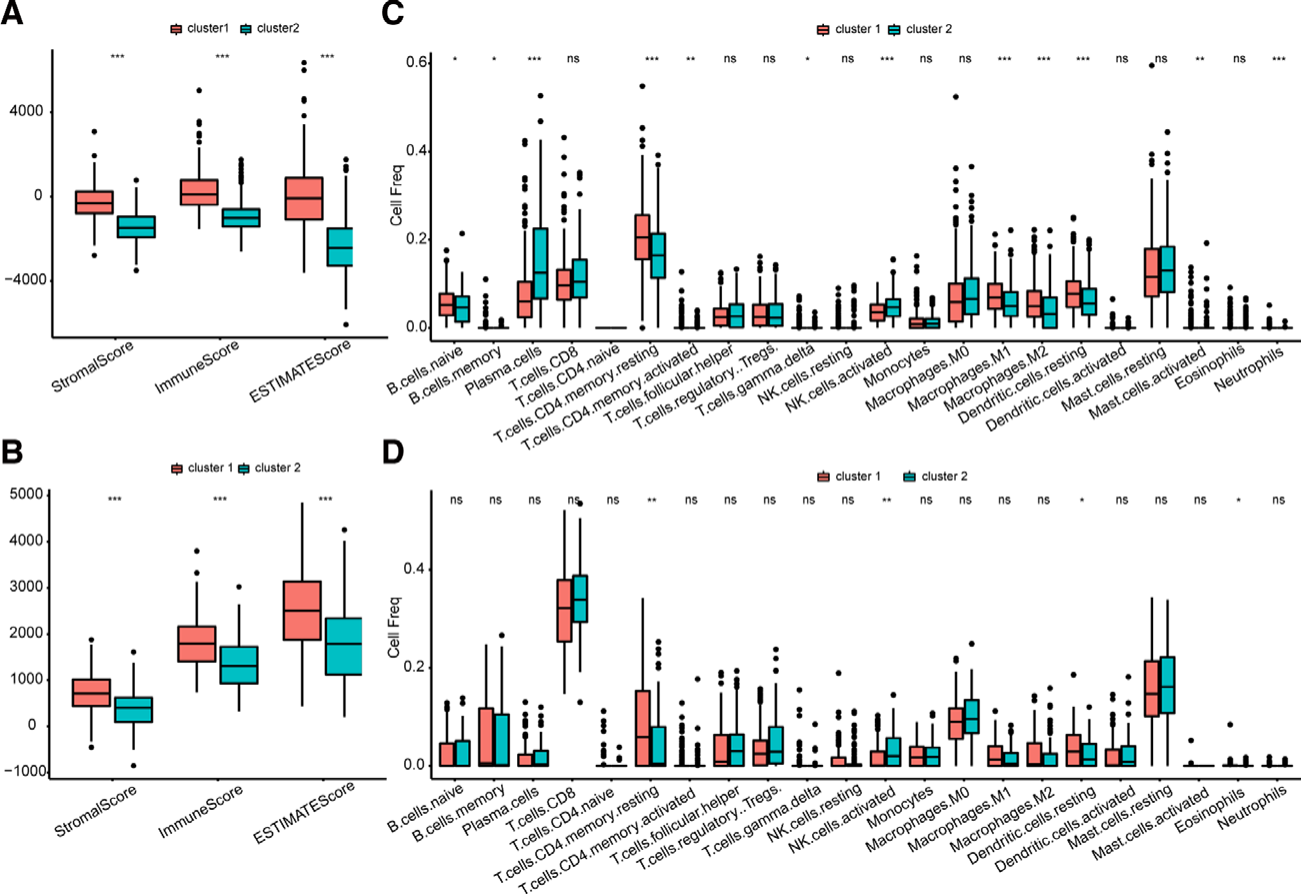
Comparison of tumor associated immune infiltration levels between the 2 clusters. (A and B) The different distributions of ImmuneScore, StromalScore, and ESTIMATEScore between the 2 clusters in the TCGA and GSE116918 datasets, respectively. (C and D) The comparison of infiltration levels of 22 immune cells between the 2 clusters in the TCGA and GSE116918 datasets, respectively. TCGA = the cancer genome atlas.

We next compared the immune infiltration levels in the 2 clusters. Notably, higher Immune, Stromal, and Estimate scores were observed in cluster 1 than in cluster 2 in the TCGA database (Fig. 3A), and consistent results were identified in the GEO database (Fig. 3B). These findings indicated that tumor-infiltration immune cell levels were different in both clusters. Therefore, further comparison of the distribution of immune cells was conducted. Consequently, significant distribution differences of many immune cells were found between the 2 clusters in the TCGA dataset (Fig. 3C), including B cells naive, B cells memory, Plasma cells, T cells CD4 memory resting, T cells CD4 memory activated, T cells gamma delta, NK cells activated, Macrophages M1, Macrophages M2, Dendritic cells resting, Mast cells activated, Neutrophils (*P* < .05). Moreover, T cells CD4 memory resting, NK cells activated, and Dendritic cells resting were differentially distributed between cluster 1 and cluster 2 in the GEO dataset (Fig. 3D).

### 3.4. Identification of CPRS in PRAD

**Figure 4.**
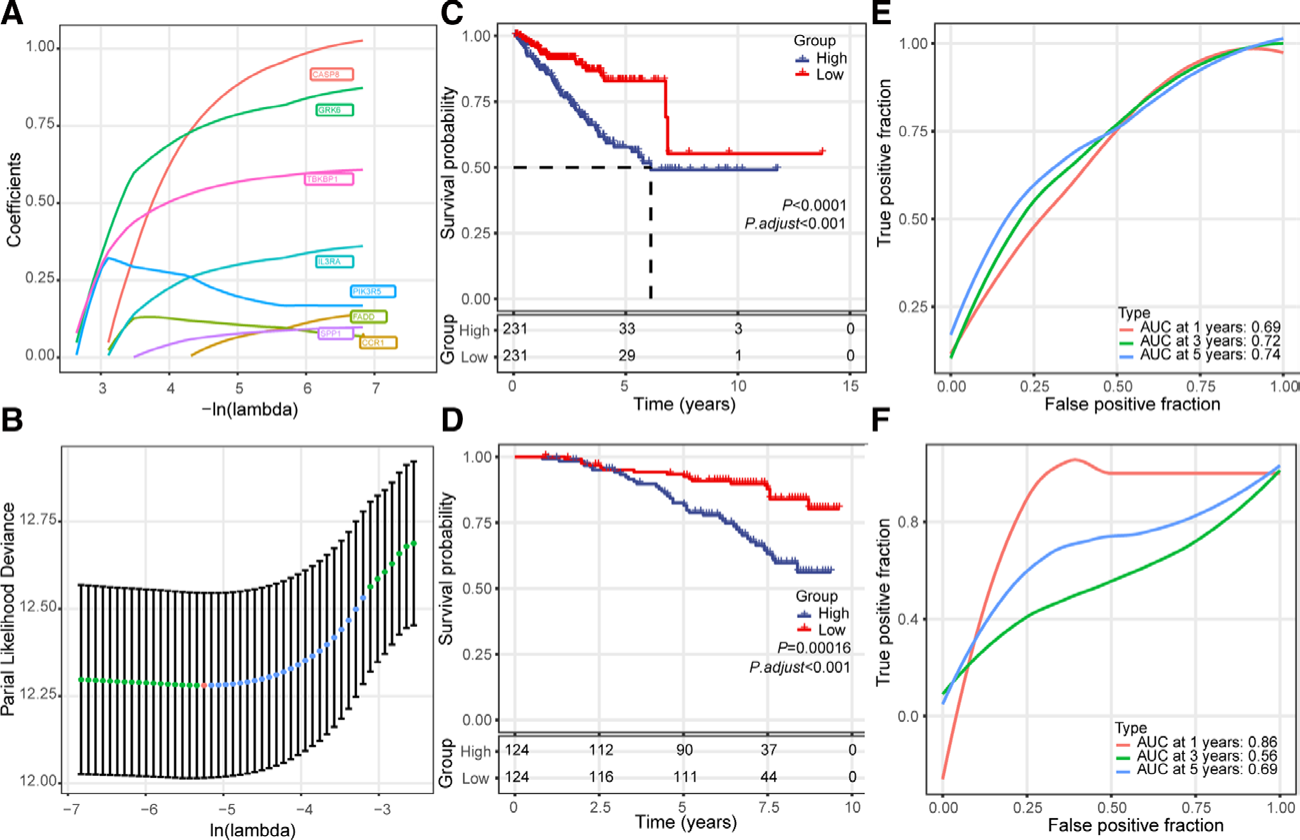
Construction and validation of the cGAS-STING pathway-related gene signature to predict PRAD patient survival. (A) Coefficients of 6 OS-related cGAS-STING pathway-associated genes in lasso Cox regression. (B) Identification of significant cGAS-STING pathway-associated genes for developing the prognostic risk score model. (C and D) Kaplan–Meier survival curves of the OS of high- or low-risk patients in TCGA cohort (C), and GSE116918 cohort (D). (E and F) Time-dependent ROC curves for 1-, 3-, and 5-yr OS by the risk score in TCGA cohort (E), and GSE116918 cohort (F). cGAS-STING = the cyclic GMP-AMP synthase-stimulator of interferon genes, lasso = least absolute shrinkage and selection operator, OS = overall survival, PRAD = prostate adenocarcinoma, ROC = receiver operating characteristic.

The 6 prognosis-related genes (*CASP8*, *GRK6*, *IL3RA*, *PLCB1*, *TBKBP1*, and *TNFSF10*) were screened as the most significant indicators, generating the CPRS to assess the patient survival in PRAD (Fig. 4A and B). The formula of CPRS is 1.287 × Exp_*CASP8*_* *+ 0.993 × Exp_*GRK6 *_+ 0.501 × Exp_*IL3RA *_− 0.596 × Exp_*PLCB1 *_+ 0.578 × Exp_*TBKBP1 *_− 0.555 × Exp_*TNFSF10*_. Based on the median values of CPRS, all patients were allocated into the high- and low-risk groups. Kaplan–Meier curves demonstrated that patients suffered a more dismal prognosis in the high-risk group than in the low-risk group in the TCGA cohort (*P* < .001, Fig. 4C). Similarly, poorer outcomes were validated in the high-risk group in the GSE116918 cohort (Fig. 4D). The predicted 1-, 2-, and 3-year survival AUC were 0.69, 0.72, 0.74 in the TCGA dataset (Fig. 4E) and 0.86, 0.56, 0.69 in the GSE116918 cohort (Fig. 4F), respectively. This evidence indicated that the CPRS was valuable and practicable in the independent dataset.

In addition, univariate and multivariate Cox regression analyses were performed to identify the prognosis-related parameters. Consequently, univariate analysis demonstrated that T stage, N stage, and CPRS were significantly related to significantly related to OS in the TCGA dataset (Hazard ratio [HR] 95% confidence interval [CI], 2.133 [1.607–2.830], *P* < .001; HR [95% CI], 1.979 [1.200–3.263], *P* = .007; HR [95% CI], 2.451 [1.457–4.121], *P* = .001, respectively) (Table S1, http://links.lww.com/MD/H700). Additionally, multivariate analysis showed that T stage and CPRS were OS-related independent clinical variables in the TCGA dataset (HR [95% CI], 2.010 [1.500–2.692], *P* < .001; HR [95% CI], 2.403 [1.408–4.101], *P* = .001; respectively) (Table S1, http://links.lww.com/MD/H700).

### 3.5. TME and immune treatment response in the 2 risk groups

**Figure 5.**
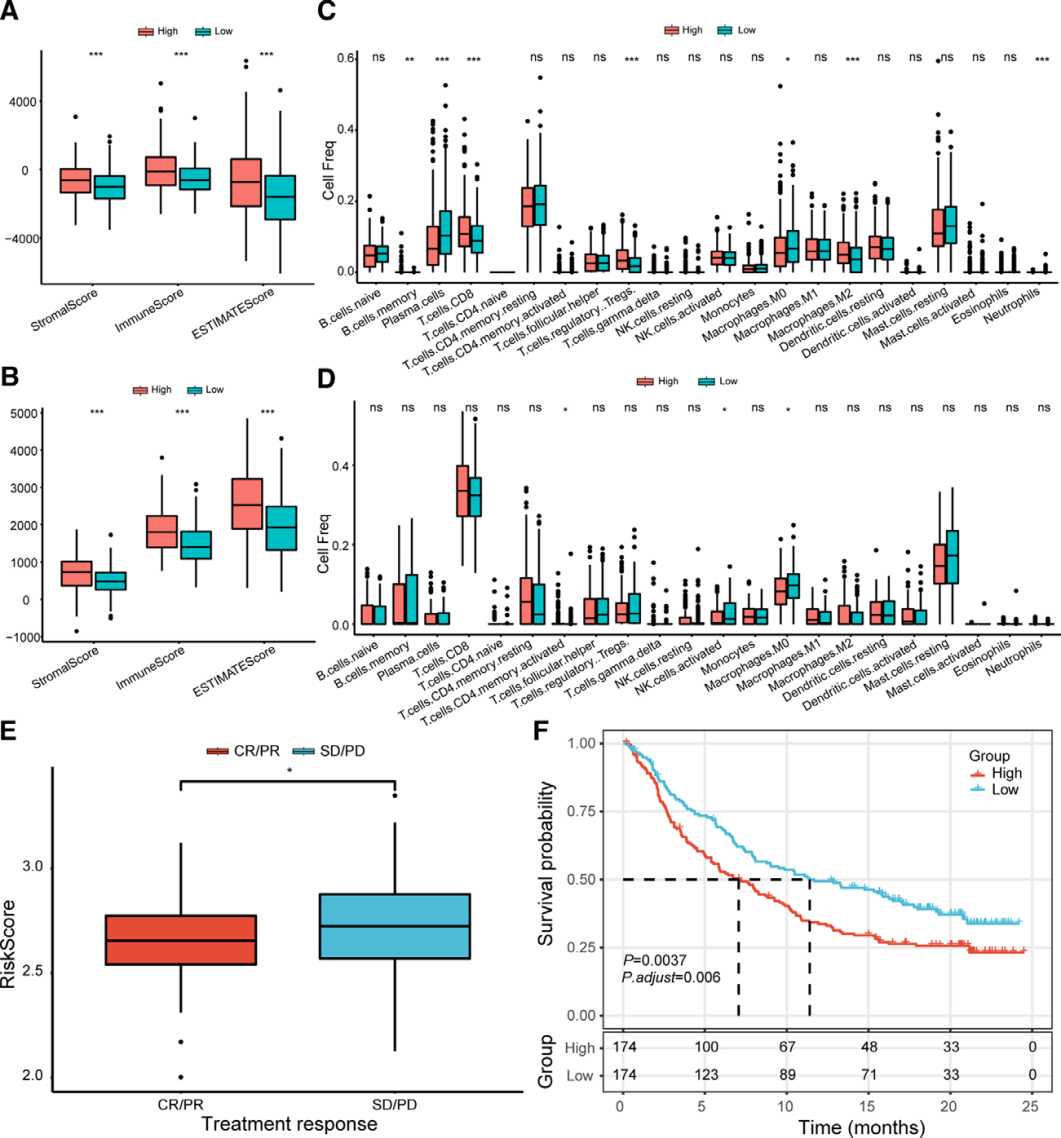
Comparison of tumor associated immune infiltration levels and immune treatment responses between the high- and low-risk groups. (A and B) The different distributions of Immune, Stromal, and ESTIMATE Scores between high- and low-risk groups in the TCGA and GSE116918 datasets, respectively. (C and D) The comparison of infiltration levels of 22 immune cells between high- and low-risk groups in the TCGA and GSE116918 datasets, respectively. (E) The relationship between the risk score and the treatment responses in the IMvigor210 database. (F) Kaplan–Meier survival curves of the OS of high- or low-risk patients in the IMvigor210 database. OS = overall survival, TCGA = the cancer genome atlas.

ESTIMATE algorithm demonstrated that higher ImmuneScore, StromalScore, and EstimatedScore were observed in the high-risk group than in the low-risk group in the TCGA dataset (Fig. 5A). Similar results were verified in the GSE116918 dataset (Fig. 5B). Moreover, patients with low risk showed an improved Macrophages M0 level compared to those with high risk in TCGA and GSE116918 datasets (Fig. 5C and D).

The IMvigor210 cohort was analyzed in this study to evaluate the potential function of CPRS in predicting immunotherapy response. The CPRS was significantly higher in the patients with stable disease or progression disease (PD) than in those with complete response or partial response (Fig. 5E). In addition, Survival analyses demonstrated a more favorable prognosis in the patients with low risk than those with high risk (*P* = .0037, Fig. 5F). These findings demonstrated that the CPRS was valuable in assessing the immunotherapy responses in patients with PRAD.

## 4. Discussion

The cGAS-STING pathway is essential in inflammation-driven tumor occurrence and progression.^[13]^ The chronic activation of downstream effector programs of cGAS-STING is associated with persistent inflammation and tumor progression.^[13,14]^ In addition, the cGAS-STING pathway is also involved in the DNA sensing, immune infiltration, and anti-tumoral immune response.^[14,15]^ The STING-targeted therapies have been considered a new class of anti-tumor immunotherapy candidates, and their safety and efficacy have been explored in multiple clinical trials (NCT02675439, NCT03010176, and NCT04144140). A recent publication demonstrated that 5 cGAS-STING pathway-related genes (*IFNB1*, *IFNA4*, *IL6*, *NFKB2*, and *TRIM25*) could be a good risk factor for predicting OS in patients with gastric cancer.^[8]^ However, to the best of our knowledge, the potential values of cGAS-STING pathway-related genes in the PRAD are still unclear, and this is the first study to detect the potential function of cGAS-STING pathway-related genes in PRAD.

This study identified 2 clusters based on the expression matrix of twelve prognosis-related genes. Specifically, patients showed better survival outcomes in cluster 2 than cluster 1 in the TCGA database. Inflammation-related pathway enrichment and immune cell infiltration levels were altered between clusters 1 and 2, suggesting different regulations of the cGAS-STING pathway between the 2 clusters. Moreover, the CPRS was developed to differentiate patients with high risk from those with low risk. Patients in the high-risk group showed a more dismal survival than those in the low-risk group in TCGA and GEO datasets. Notably, the CPRS can also differentiate responsive patients from non-responsive individuals treated with PD-L1 blockades. In addition, patients with high CPRS demonstrated a more favorable prognosis than those with low CPRS. These findings indicated that CPRS was a valuable indicator for survival prediction and immunotherapeutic response assessment in patients with PRAD. Closer follow-up plans and appropriate strategy-related decisions can be made in PRAD patients with high CPRS.

The proposed CPRS comprises 6 prognosis-related genes, including *CASP8*, *GRK6*, *IL3RA*, *PLCB1*, *TBKBP1*, and *TNFSF10*. As a caspase protein, CASP8 is essential in apoptosis, necroptosis, and pyroptosis.^[16]^ Improved mRNA levels of CASP8 were identified in the patients with recurrent prostate cancer compared with those with non-recurrent prostate cancer.^[17]^ Prognostically, enhanced expression levels of CASP8 were correlated with dismal disease-free survival and OS in renal cancer.^[17]^ GRKs are a versatile family of protein kinases. GRK6 has been reported as a potential biomarker enriched in prostate cancer cell exosomes.^[18]^ Aberrant expression of GRK6 was associated with dismal prognosis in multiple cancer types.^[19–21]^ Another prognosis-related gene, IL3RA, is highly expressed on the surface of multiple cells. It is also an essential biomarker and promising immunotherapeutic target in hematologic malignancies.^[22,23]^ PLCB1 involved tumorigenesis in various cancer types, such as hepatocellular carcinoma, cholangiocarcinoma, and non-small cell lung carcinoma.^[24–26]^ This finding suggested that it could participate in the tumor development and progression in the PRAD. A recent study reported that TBKBP1 and TBK1 generate a growth factor signaling axis, mediating immunosuppression and tumorigenesis.^[27]^ As one of the members of the TNF family, TNFSF10 can induce cell apoptosis in multiple cancer types, including prostate cancer.^[28,29]^ These findings indicated that 6 screened prognosis-related genes could impact tumorigenesis and progression functionally, helping us better understand their values in survival prediction and immunotherapeutic response assessment.

Immunotherapy is one of the most significant breakthroughs in cancer treatment.^[30]^ Combining PD-1/PD-L1 blockades with other anti-tumor therapies has yielded impressive clinical efficacy in prostate cancer.^[31]^ The activation of the cGAS-STING pathway impacts multiple steps in the cancer-immunity cycle,^[15]^ and it can mediate onco-suppressive effects through induction of cancer cell senescence.^[32]^ Upregulation of STING was associated with improved infiltration levels of regulatory T cells and expression levels of immunoregulatory enzyme indoleamine 2,3-dioxygenase, which involved tumor immune evasion and inhibition of T cell proliferation.^[33]^ In this study, we found that the distribution of immune cells was different in the 2 clusters and 2 risk groups. Moreover, the CPRS can stratify high- or low-risk patients and predict immunotherapeutic responses. These findings indicated the robust predictive performance of CPRS in survival and immunotherapeutic assessment.

There are several limitations to this study. First, although the CPRS has been considered a promising and practicable prognostic indicator in patients with PRAD, its prognostic value still needs to be verified in a prospective study. Next, to further understand the mechanisms of these components of CPRS, functional studies should be performed to verify the potential crosstalk between these predictors and the cGAS-STING signal pathway in the future.

In conclusion, the proposed risk model was developed based on the cGAS-STING pathway related-genes and can be used as a promising predictor for patient survival in PRAD. Moreover, it can also stratify the responded and non-responded patients who received anti-PD-L1 agents, which may help clinicians better assess treatment response and survival, contributing to treatment strategy-related decision-making.

## Acknowledgments

We acknowledge TCGA and GEO databases for providing their platforms and contributors for uploading their meaningful datasets.

## Author contributions

Conception and design: Y.S.; Administrative support: All authors; Provision of study materials: All authors; Collection and assembly of data: Z.X.X.; Data analysis and interpretation: Z.X.X.; Manuscript writing: All authors; Final approval of manuscript: All authors.

Conceptualization: Sui Yu.

Data curation: Xingxing Zhuo.

Formal analysis: Xingxing Zhuo, Sui Yu.

Methodology: Xingxing Zhuo, Hao Dai, Sui Yu.

Project administration: Xingxing Zhuo.

Software: Xingxing Zhuo.

Validation: Xingxing Zhuo.

Writing – original draft: Xingxing Zhuo, Hao Dai, Sui Yu.

Writing – review & editing: Xingxing Zhuo, Hao Dai, Sui Yu.

## Supplementary Material
