# Supplementary figures and images for: The cGAS-STING pathway-related gene signature can predict patient prognosis and immunotherapy responses in prostate adenocarcinoma
Source: Medicine (Baltimore). 2022 Dec 16;101(50):e31290. doi: 10.1097/MD.0000000000031290 (PMC9771290; doi:10.1097/MD.0000000000031290)

Supplementary Figure 1

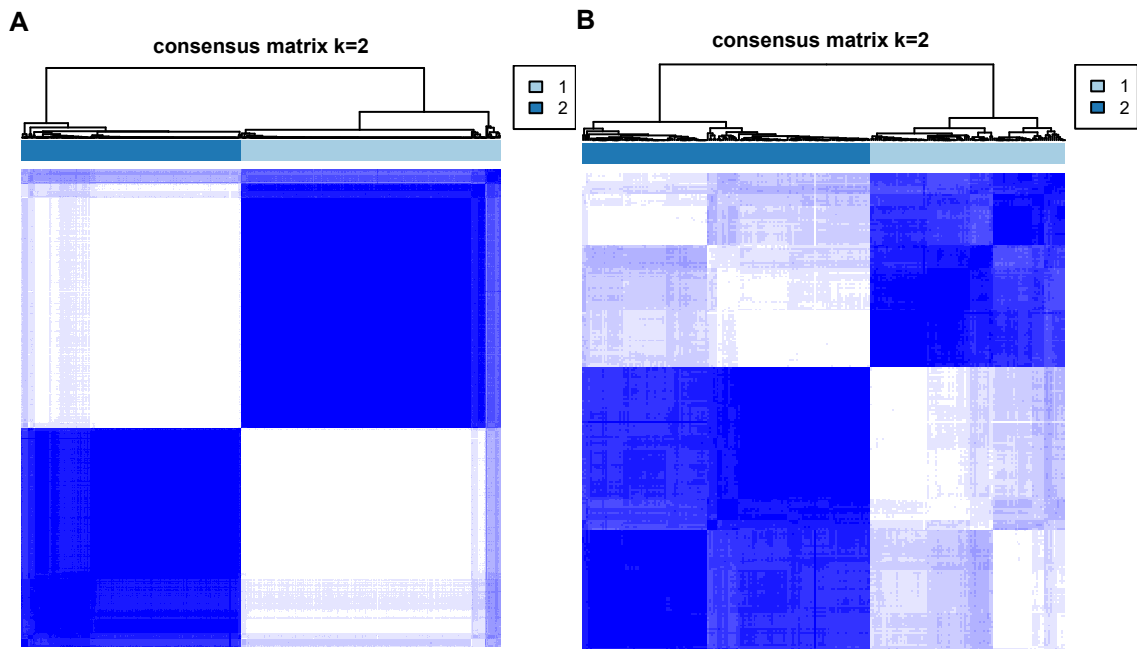

Supplement: Supplementary file 1 [file medi-101-e31290-s001.pdf]
