# Supplementary material for: The cGAS-STING pathway-related gene signature can predict patient prognosis and immunotherapy responses in prostate adenocarcinoma
Source: Medicine (Baltimore). 2022 Dec 16;101(50):e31290. doi: 10.1097/MD.0000000000031290 (PMC9771290; doi:10.1097/MD.0000000000031290)
Supplement: Supplementary file 2 [file medi-101-e31290-s002.pdf]

Supplementary Table 1. Univariate and multivariate Cox regression analysis based on different clinical characteristics and OS in patients with PRAD.

Characteristics	Univariate model		Multivariate model	
	HR 95% CI	*P* value	HR 95% CI	*P* value
Age	1.026 (0.993-1.061)	0.129		
T stage	2.133 (1.607-2.830)	<0.001	2.010 (1.500-2.692)	<0.001
N stage	1.979 (1.200-3.263)	0.007	1.303 (0.767-2.213)	0.328
M stage	0.000 (0.000-Inf)	0.996		
CPRS	2.451 (1.457-4.121)	0.001	2.403 (1.408-4.101)	0.001
